# Factors associated with prolonged hospital stay of imported malaria cases in Chengdu, China: a retrospective study

**DOI:** 10.1186/s12879-022-07464-6

**Published:** 2022-05-26

**Authors:** Liang Huang, Hong Jin, Hong Zhang, Yang Liu, Xinxing Shi, Xintong Kang, Yilan Zeng, Lin Wang

**Affiliations:** 1 Chengdu Public Health Clinical Center, Chengdu City, 610000 Sichuan Province China; 2 Sichuan Center for Disease Control and Prevention, Chengdu City, 610000 Sichuan Province China

**Keywords:** Malaria, Prolonged hospital stay, Imported cases

## Abstract

**Background:**

Although China has entered the post-malaria-elimination era, imported cases remain a public health concern in China.

**Methods:**

We retrospectively analyzed data from cases of imported malaria from January 2017 to December 2020 in Chengdu Public Health Clinical Center. We assessed potential clinical, epidemiological, geographical, and seasonal effects on duration of hospital stay. Cox proportional hazards model was used to identify predictive factors for prolonged hospital stay. Multivariate logistic regression was used to assess the potential risk factors associated with severe cases.

**Results:**

The highest number of imported cases of malaria were from the Democratic Republic of the Congo (23%, 34/150) and most patients (74%, 26/34) were infected by *Plasmodium falciparum*. The Edwards test indicated no significant seasonality in imported cases of malaria (χ^2^ = 2.51, *p* = 0.28). Bacterial infection (adjusted hazard ratio [aHR] for discharge = 0.58, *p* = 0.01) and thrombocytopenia (aHR = 0.66, *p* = 0.02) were risk factors for prolonged hospital stay. The C-reactive protein (OR = 1.02, *p* = 0.01) and procalcitonin (OR = 1.03, *p* = 0.01) were risk factors for severe cases.

**Conclusions:**

Bacterial infection and thrombocytopenia are risk factors for prolonged hospital stay among imported malaria cases. The C-reactive protein and procalcitonin level were risk factors for severe cases.

## Background

Following implementation of the National Malaria Elimination Program (NMEP) in 2010 [1], China reported its final indigenous case of malaria in 2015 in Canyuan County, Yunnan Province [2]. In 2017, after three consecutive years of zero indigenous malaria cases reported [3], China fulfilled the World Health Organization malaria elimination criteria and entered the post-elimination era [4]. Since that time, all malaria cases have been imported from overseas, mostly by returning Chinese workers. The steadily increasing number of imported malaria cases poses a significant public health risk to Chinese society [5, 6].

Chengdu City is the provincial capital of Sichuan province, which occupies much of Western China including the eastern Qinghai-Tibet plateau. Chengdu Public Health Clinical Center (CPHCC) is the only tertiary care center for infectious diseases in Chengdu City and provides services for much of Sichuan province. Most imported malaria patients in Sichuan province are hospitalized in CPHCC. In 2019, CPHCC passed the national examinations for the NMEP; requirements include standards for malaria patient management, disease reporting, treatment, diagnosis, laboratory equipment, and staff training. Thus, data from patients with imported malaria in CPHCC may be considered representative of imported malaria in China.

Treatments for malaria are effective only if administered in a timely fashion [7, 8]. However, our experience has been that the clinical conditions of patients with imported malaria and length of hospital stay (LOS) varies widely. In this study, we investigated predictive factors for prolonged hospital stay among patients with imported malaria to provide evidence to inform clinical decision-making and patient management. No previous study has covered this topic among imported malaria cases in China. We although assessed the potential factors associated with severe cases.

## Methods

### Patients and data source

We retrospectively analyzed data for imported malaria patients admitted in CPHCC from Jan. 2017 to Dec. 2020. All the patients firstly went to emergency department or fever clinic of CPHCC, many of them referred by other hospitals. A total of 150 cases were enrolled. Demographic and clinical data were collected, including medical history, main complaints, and source country. Laboratory parameter results included routine blood tests, liver function tests, and medical imaging records.

Malaria was confirmed in all patients by pathologic evidence of *Plasmodium* parasites. All confirmed cases were included in this study. Patients with suspected malaria who were subsequently diagnosed with other conditions by additional medical investigations were excluded from the study.

Ethical approval for this study was obtained from the Chengdu Public Health Clinical Center Ethics Committee. The requirement for informed consent was waived because of the retrospective nature of the study and the data were anonymous.

### Criteria for diagnosis and discharge

Malaria diagnosis was based on national recommendations for malaria diagnosis (WS 259-2015) [9].

The criteria for discharge were based on the rules of CPHCC as follows: (1) no evidence of *Plasmodium* parasites in the blood by microscopy of blood smears; (2) significant improvement in clinical signs, manifestations, and laboratory examinations; and (3) normal body temperature for more than 3 days.

### Statistical analysis

Statistical analyses were performed using STATA/SE 14.1 software (StataCorp, College Station, TX, USA) and R version 4.0.2 [10]. The SEAST module for STATA was installed to implement the Edwards seasonality test [11]. The R package networkD3 was used for diagram plotting [12].

Normally distributed continuous data were presented as means and standard deviations and non-normally distributed continuous data were presented as medians and ranges. Multivariable Cox proportional hazard regression and Multivariate logistic regression with stepwise variable selection were used to find the potential factors associated with prolonged hospital stay and severe cases. The Edwards test for seasonality was used for time series case number data [11]. The log-rank test was used to test for equality of survivor functions. Values of *p* < 0.05 were considered statistically significant.

## Results

### General characteristics of patients

All patients were Chinese nationals and acquired malaria infection outside China. All patients were providing labor services in Africa or two Asian countries (Myanmar and Pakistan). All were male with a median age of 42 years (range: 19–62 years). All were in good health and had undergone physical examinations before going abroad (Table 1).

**Table 1:** General characteristics of patients with imported malaria in Chengdu, China (n = 150)

Variables	Mean/Median/%	SD/Range
Age (years)	40.18	11.01
Days of Fever before admission (Days)	3	0.2–30
Previous infection times (times)	1	1–11
Parasite density (%)^1^	0.2%	0–15%
Bacterial infection (%)	20%	–
Length of stay (Days)	7	3–23
ALT (U/L)	38	7–199
AST (U/L)	38	14–235
TBIL (μmol/L)	19.60	5–334.7
WBC (10^9^/L)	5.43	1.58–11.15
PLT (10^9^/L)	84	10–624
HGB (g/L)	135	77–177
ALB (g/L)	37.95	21.6–50.6
Creatinine (μmol/L)	72	32.8–230
CRP (mg/L)	58.42	0.5–281.2

SD, standard deviation; ALT, alanine aminotransferase; AST, asparagine aminotransferase; TBIL, total bilirubin; WBC, white blood cell; PLT, platelet; HGB, hemoglobin; ALB, albumin; CRP, C-reactive protein

^1^Percentage of infected red blood cells

### Distribution of clinical signs and symptoms

Fever is the major manifestation of malaria. Almost all patients (99%, 149/150) reported fever with varying duration (median 3 days, range 0.2–30 days). Most patients (83%, 124/150) reported shivering associated with fever, while roughly half (46%, 69/150) suffered from headaches. Other signs and symptoms included cough (19%, 28/150), productive cough (54%, 15/28), fatigue (44%, 66/150), anorexia (17%, 26/150), and muscular soreness (15%, 22/150).

### Patient medical history

The Hospital Information System (HIS) recorded details of each patient’s medical history as well as onset of malaria. Half of all patients (50%, 75/150) had been infected by *Plasmodium* species parasites twice or more, and 68% of patients had been infected at least once. Of note, 17% of patients (25/150) had been misdiagnosed at other medical facilities. Because the clinical signs of malaria are non-specific, physicians in non-endemic areas have limited experience with this diagnosis. The DOF ranged from 0.2 to 30 days with a median of 3 days.

### Geographical and pathogen distribution

Except for one patient who returned from Myanmar with *P. vivax* infection and three patients who returned from Pakistan with *P. vivax* infection, all cases originated in Africa. Most patients were infected (74%, 111/150) or co-infected (4%, 6/150) by *P. falciparum*. The second most common *Plasmodium* species was *P. vivax*, infecting and co-infecting 16% (24/150) and 3% (4/150) of patients, respectively.

**Fig. 1:**
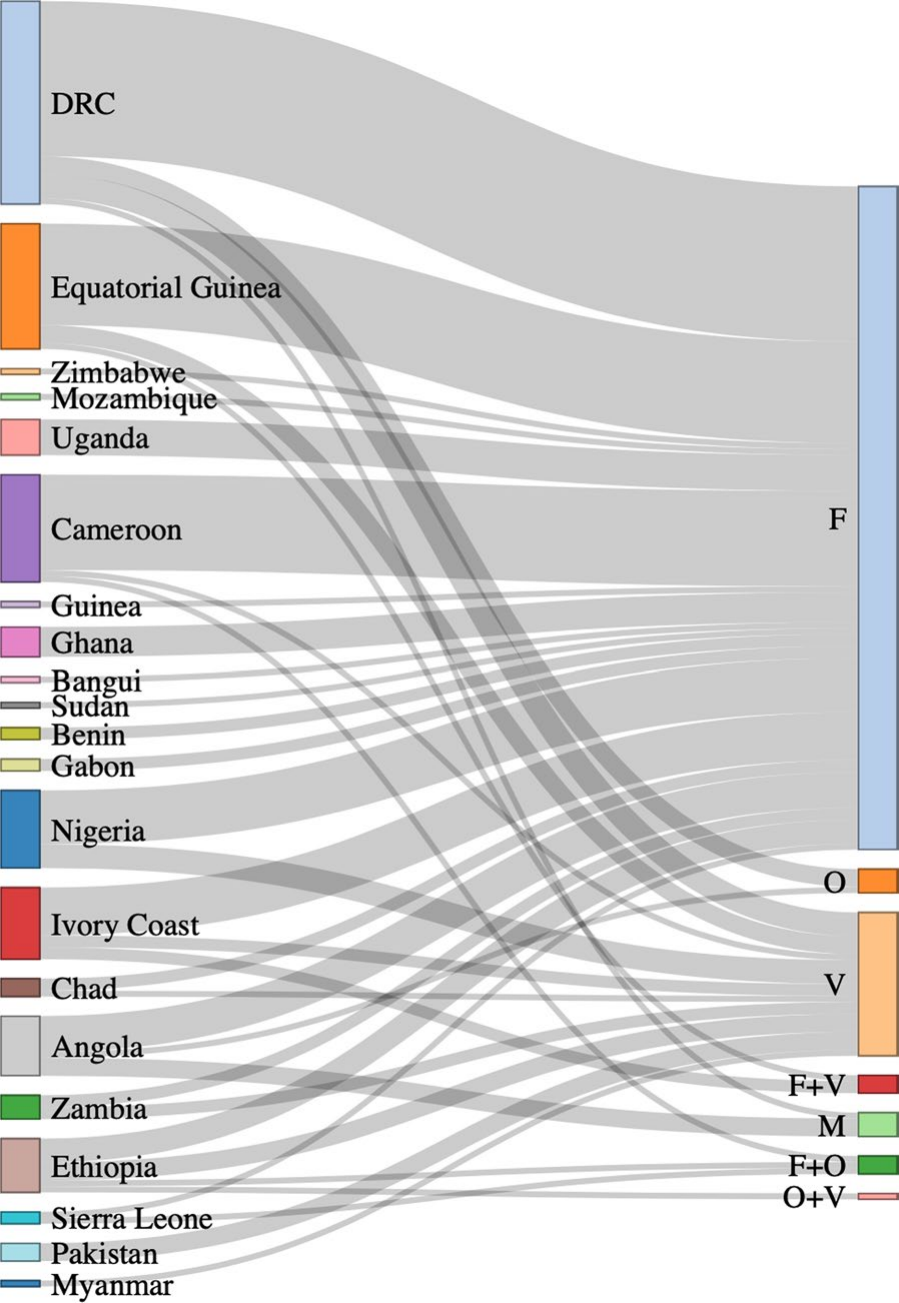
Geological and pathogen distributions for cases of imported malaria in Chengdu, China. F: *P. falciparum;* O: *P. ovale*; V: *P. vivax*; F + V: *P. falciparum* + *P. vivax*; M: *P. malariae;* F + O: *P. falciparum* + *P. vivax*; O + V: *P. ovale* + *P. vivax*. An interactive version of this diagram is available at: https://rpubs.com/hyuangx/sgview

Geographically, the highest number of imported cases of malaria were from the Democratic Republic of the Congo (23%, 34/150); approximately three-quarters of these patients (74%, 26/34) were infected by *P. falciparum*. The second-highest number of imported cases of malaria originated in Equatorial Guinea (14%, 21/150); 81% (17/21) of these patients were infected by *P. falciparum* (Tables 2 and 3)*.* A Sankey diagram was used to describe the relationship between source countries and infecting pathogens (Fig. 1).

**Table 2:** Distribution of pathogens responsible for imported malaria in Chengdu, China

Pathogens	Number of patients (%)
*P. falciparum*	111 (74.0%)
*P. ovale*	4 (2.7%)
*P. vivax*	24 (16.0%)
*P. falciparum* + P. ovale^*^	3 (2.0%)
*P. malariae*	4 (2.7%)
*P. falciparum* + P. vivax^*^	3 (2.0%)
*P. ovale* + P. vivax^*^	1 (0.7%)

*Co-infections

**Table 3:** Distribution of source countries for cases of imported malaria in Chengdu, China

Countries	Numbers of patients (%)
Nigeria	13 (8.7%)
DRC*	34 (22.7%)
Pakistan	3 (2.0%)
Equatorial Guinea	21 (14.0%)
Myanmar	1 (0.7%)
Zimbabwe	5 (3.3%)
Mozambique	1 (0.7%)
Ivory Coast	12 (8.0%)
Uganda	6 (4.0%)
Guinea	1 (0.7%)
Cameroon	18 (12.0%)
Chad	3 (2.0%)
Ethiopia	9 (6.0%)
Ghana	5 (3.3%)
Bangui	1 (0.7%)
Sudan	1 (0.7%)
Angola	10 (6.7%)
Benin	2 (1.3%)
Gabon	2 (1.3%)
Sierra Leone	2 (1.3%)

*DRC, Democratic Republic of the Congo

### Time distribution of cases of imported malaria

**Fig. 2:**
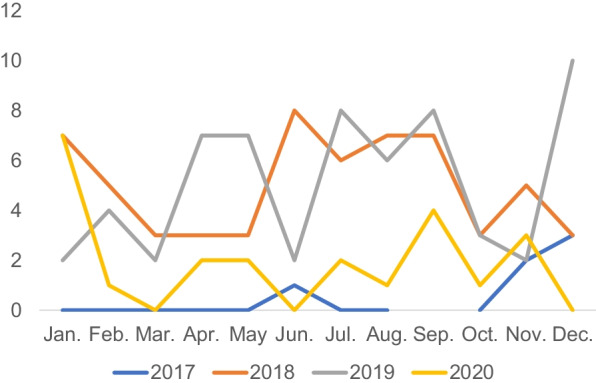
Time distribution of cases of imported malaria in Chengdu, China. X-axis shows the month of admission. Y-axis shows the number of cases

Cases of imported malaria over the four years of our study were plotted (Fig. 2). No significant variation over time was observed. A decrease in imported malaria cases in 2020 likely resulted from COVID-19 public health measures.

To assess whether cases of imported malaria at CPHCC depended on season, we used the Edwards test for seasonality [11]. Based on the result of this test (χ^2^ = 2.51, *p* = 0.28), there appeared to be no significant seasonality in imported malaria cases at CPHCC (Fig. 2).

### Laboratory tests in patients with imported malaria

All patients with malaria underwent emergency medical evaluations regardless of clinical condition or potential prognosis. Assessments included routine blood tests, blood biochemistry, microscopy of blood smears, and *Plasmodium* rapid diagnostic tests (RDT) [13], X-ray or computed tomography (CT) scans of the lung, and in most cases procalcitonin (PCT) and C-reactive protein (CRP) examinations.

Decreased platelet (PLT) counts were observed in most patients: the median PLT count was 84 × 10^9^/L (range: 10–624 × 10^9^/L). Only 3% (5/150) of patients had white blood cell (WBC) counts higher than 10 × 10^9^/L (median: 5.43 × 10^9^/L, range: 1.58–17.15 × 10^9^/L). However, 22% (33/150) of patients had WBC counts lower than 4 × 10^9^/L.

Mild liver injury is common among patients with malaria. In the present study, 13% (20/150) of patients had alanine aminotransferase (ALT) levels higher than 2 × the upper limit of normal (37 U/L). ALT levels ranged 9 to 199 U/L with a median of 38 U/L. Hypoproteinemia (ALB < 35 g/L) was present in 31% (46/150) of patients. The median of ALB was 37.95 g/L, ranged 21.6–50.6 g/L.

Bacterial infections are major complications of malaria. In the present study, 20% (30/150) of patients were diagnosed with bacterial infections. All except one (97%, 29/30) were bacterial pneumonia (one patient was diagnosed with a urinary tract infection). Diagnosis of bacterial infection was made by CT scans or X-ray imaging, clinical manifestations, and other relevant examinations.

Microscopy of blood smears showed that 8% (12/150) of patients had high-density infection (≥ 5% red blood cells infected).

### Predictive factors for prolonged hospital stay

The median LOS was 7 days (range: 3–23 days). We defined the failure event as discharge following medical advice (no patients were discharged against medical advice). The time variable used in Cox model was the LOS. Thus, aHRs (Adjusted Hazard Ratios) were for risk of discharge and LOS was prolonged for aHRs < 1. The independent variables included pathogen type, elder (> 50 years old), bacterial infection, abnormal liver function (ALT > 37U/L), low PLT count (< 100 × 10^9^/L), leukocytopenia (< 4.0 × 10^9^/L), anemia (< 120 g/L), jaundice (total bilirubin > 20 µmol/L), high density infection (≥ 5% red blood cells infected), long DOF (> 5 days), misdiagnosis, hypoproteinemia (albumin < 35 g/L), PCT (procalcitonin), and CRP(C-reactive protein) were included in the full Cox model.

Laboratory abnormalities were defined by criteria of CPHCC. The high density infection was defined as parasitemia of ≥ 5% red blood cells. Those variables were common laboratory examinations carried out for each malaria patients.

**Fig. 3:**
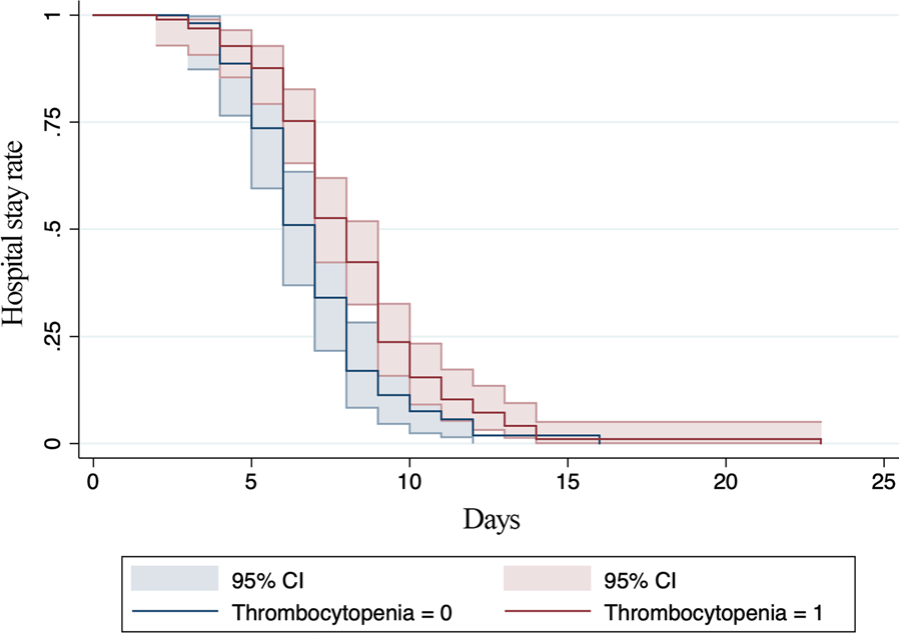
Survival functions for patients with imported malaria in Chengdu, China with and without thrombocytopenia. *CI* confidence interval

**Fig. 4:**
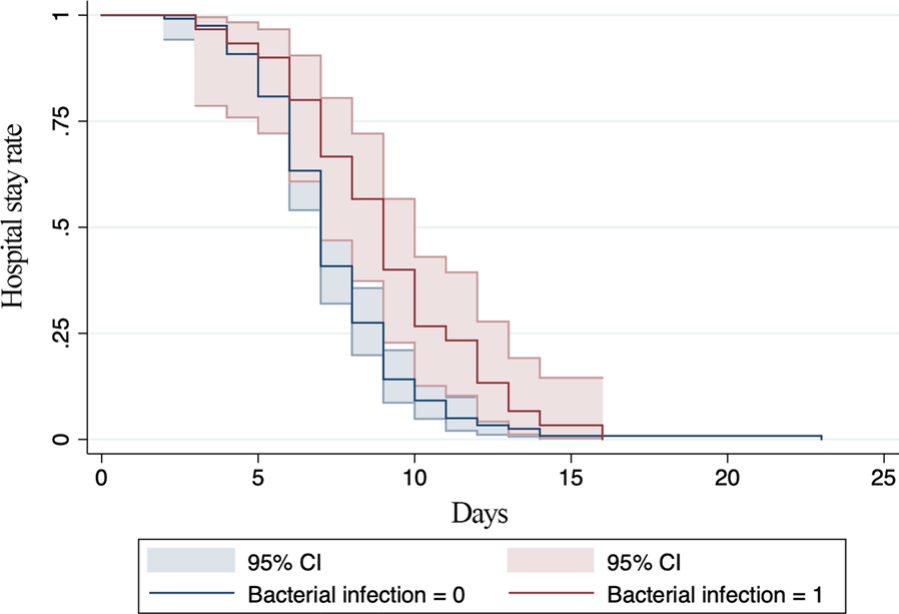
Survival functions for patients with imported malaria in Chengdu, China with and without bacterial infections. *CI* confidence interval

Using stepwise variable selection, bacterial infection and thrombocytopenia were statistically significant (aHRs for discharge 0.58 [*p* = 0.01, 95% confidence interval (CI) 0.38–0.88] and 0.66 [*p* = 0.02, 95% CI 0.47–0.94], respectively). The results are shown in Table 4. The survival functions are shown in Figs. 3 and 4.

**Table 4:** Cox proportional hazard model to identify predictors of prolonged hospital stay among patients with imported malaria in Chengdu, China

Variables	aHR	SE	z	95% CI		*p*-value
Bacterial infection	0.58	0.12	− 2.59	0.38	0.88	0.01
*P. falciparum* infection solely	4.93	2.62	3.00	1.74	13.98	0.00
Leukopenia	0.66	0.14	− 1.96	0.44	1.00	0.05
Thrombopenia	0.66	0.12	− 2.32	0.47	0.94	0.02

*HR* hazard ratio, *SE* standard error, *CI* confidence interval

*P. falciparum* infection was associated with discharge (aHR 4.93, 95% CI 1.74–14.00) and thus protective against prolonged LOS, because the antimalarial agent was ACTs normally. The ACTs have shorter course of treatments.

Log-rank testing of the survivor functions for bacterial infection and hypoproteinemia were statically significant (χ^2^ values 8.0 and 8.16, respectively; both *p* < 0.001) (Figs. 3 and 4).

### Severe cases and major complications

Nine cases (6%, 9/150) were severe cases in this study. The median LOS was 10 days (ranged 6–14 days) which was significantly longer than other cases (z = − 2.75, *p* = 0.00). To identify the potential factors associated with severe cases, the multivariate logistic regression with stepwise variable selection was used. The independent variables were same with Cox proportional hazard model (previous section). The multivariate logistic regression revealed that concentration of CRP was a risk factor for severe cases (OR = 1.02, 95% CI 1.00–1.04, *p* = 0.02). The PCT was identified as a risk factor for severe cases as well (OR = 1.03, 95% CI 1.01–1.06, *p* = 0.01). Five patients in the nine severe cases had complication of cerebral malaria.

### Treatments and outcomes

All treatments complied with national recommendations for malaria treatments (WS/T 485-2016) [14]. The antimalarial agent selections were majorly depending on the type of *Plasmodium* parasites. ACTs were used for *P. falciparum* infection. Chloroquine + primaquine were used for *P. vivax* or *P. ovale* infections. Artemether or artesunate were used for severe cases. In our study, all patients fulfilled the discharge criteria and no patients died.

## Discussion

### Risk of malaria in the post-elimination era in China

Malaria prevention efforts should target laborers working abroad given the increasing number of workers returning from overseas [6]. A major challenge in this regard is lack of awareness of malaria among physicians at primary medical care facilities [15]. In our study, patients spent median of 3 days (DOF ranged 0.2–30 days) before presentation in our center, 17% (25/150) of cases were misdiagnosed at other medical facilities. Thus, there is a continued need for training of clinicians regarding signs and symptoms of malaria in the post-elimination era.

However, misdiagnosis and long DOF (> 5 days) were not associated with prolonged LOS as shown by Cox proportional hazard modeling.

### Predictive factors for prolonged hospital stay and risk factors for severe cases

Through Cox proportional hazard modeling, we found the low PLT counts and bacterial infection were predictive factors of prolonged hospital stay. Prolonged hospital stays imply a more serious clinical situation, with longer time required to fulfill the discharge criteria. Patients with longer LOS also consume more medical resources and introduce higher local transmission risks. A previous study also found that PLT count was predictive of the severity of imported malaria [16]. Varadaraj reported that 97.6% (82/84) of patients with malaria had thrombocytopenia [17]. Due to the above reasons, we included the thrombocytopenia in the models in this study.

Bacterial infections are common in malaria patients [18]. A systematic review found that 6.5% of African children with *P. falciparum* infection also had bacterial infections [19]. In our study, nearly all patients with bacterial infections had bacterial pneumonia (one had a urinary tract infection) and were successfully treated with antibiotics. However, in some patients bloodstream infection may occur and cause serious problems [20].

The previous studies indicated the CRP was a prognostic factor and a biomarker for malaria infection and monitoring of malaria severity [21, 22]. PCT levels were reported as biomarkers for severe *P. falciparum* disease [23]. In our study, the multivariate logistic regression verified independently that PCT and CRP were associated with severe case.

### Seasonal patterns of malaria

We found no evidence of seasonality in the 150 imported malaria cases admitted to our hospital. However, previous studies revealed a seasonal pattern of malaria incidence in Burundi [24]. Another study indicated seasonal transmission of malaria in Niger [25]. Mbouna et al. found seasonal relationships between climate, population density, and malaria indicators in Cameroon [26]. We conclude that the incidence of malaria in Africa does not directly affect infection rates among returning laborers, whose travel is normally pre-planned. This finding indicated that hospitals should prepare special malaria wards regardless of seasonal variations of disease incidences in endemic areas. In our hospital the malaria wards were isolated from those for other diseases and were equipped with mosquito control measures to minimize the possibility of local transmission.

### Clinical implications of geographical and pathogen distribution

Understanding the geographical distribution of pathogens is important for clinical diagnosis and treatment. If clinicians can deduce the causative pathogens of infection based on the regions patients have visited without waiting hours for laboratory testing results, treatment may be initiated more rapidly. A review confirmed that rapid treatment initiation reduced the risk of severe disease [27, 28]. In our study, most cases of imported malaria originated from the Democratic Republic of the Congo, and these patients were infected by almost all *Plasmodium* species. However, *P. falciparum* was the most common pathogen (76%, 26/34).

## Conclusions

We studied the epidemiological and clinical features of patients with imported malaria, which represent the bulk of malaria infections in the post-elimination era in China. Misdiagnosis at other medical facilities was common, and efforts to improve this situation must be taken. Thrombocytopenia and bacterial infection were predictive factors for prolonged hospital stay. No seasonality was observed for imported malaria cases. The higher PCT and CRP associated with higher risk of severe cases.

## Data Availability

The data supporting the findings of this study are available from the corresponding author LW on request.

## Abbreviations

ALT: Alanine aminotransferase
AST: Aspartate aminotransferase
PLT: Platelet
GLB: Globulin
ALB: Albumin
TBIL: Total bilirubin
WBC: White blood cell
SE: Standard error
CI: Confidential interval
PCT: Procalcitonin
CRP: C-reaction protein
RDT: Rapid diagnostic tests
ACT: Artemisinin-based combination therapy
OR: Odds ratio
HR: Hazard ratio
